# From spine to heart: a case report of massive cement embolism following vertebroplasty

**DOI:** 10.1093/ehjcr/ytag263

**Published:** 2026-04-15

**Authors:** Eglė Majauskienė, Agnė Drąsutienė, Kamilė Leketaitė, Sigita Glaveckaitė, Ernestas Dvinelis

**Affiliations:** Clinic of Cardiovascular Diseases, Institute of Clinical Medicine, Faculty of Medicine, Vilnius University, Santariskiu str. 2, 08406 Vilnius, Lithuania; Clinic of Cardiovascular Diseases, Institute of Clinical Medicine, Faculty of Medicine, Vilnius University, Santariskiu str. 2, 08406 Vilnius, Lithuania; Faculty of Medicine, Vilnius University, M. K. Ciurlionio str. 21, LT-03101 Vilnius, Lithuania; Clinic of Cardiovascular Diseases, Institute of Clinical Medicine, Faculty of Medicine, Vilnius University, Santariskiu str. 2, 08406 Vilnius, Lithuania; Clinic of Cardiovascular Diseases, Institute of Clinical Medicine, Faculty of Medicine, Vilnius University, Santariskiu str. 2, 08406 Vilnius, Lithuania

**Keywords:** Cardiac embolism, Pulmonary cement embolism, Vertebroplasty, Pulmonary hypertension, Cardiovascular imaging, Case report

## Abstract

**Background:**

Percutaneous vertebroplasty is a commonly used, minimally invasive therapeutic intervention to relieve pain caused by vertebral compression fractures, which are becoming more prevalent due to the aging population. Although the procedure is generally safe, complications such as cement leakage into the bloodstream can occur.

**Case summary:**

We present the case of a 67-year-old female with a history of advanced osteoporosis and multiple vertebroplasty procedures who developed dyspnoea 5 months after her most recent surgery. Imaging revealed an intracardiac cement embolism extending from the inferior vena cava through the right atrium and tricuspid valve into the right ventricle. This rare complication of vertebroplasty, caused by cement leakage, resulted in tricuspid valve regurgitation and pulmonary hypertension.

**Discussion:**

This case highlights the importance of recognizing and managing the rare complications of vertebroplasty by using a multimodality imaging approach, especially in patients with a history of multiple procedures.

Learning points

- Dyspnoea following vertebroplasty is an important clinical warning sign, as it may indicate intracardiac or pulmonary cement embolism—a potential early or late complication.
- Evaluation of cardiac masses requires a multimodal imaging approach, and 3D echocardiography is a valuable, yet underutilized, component of this assessment.
- Cardiac cement embolization management options depend on symptom severity and the location of the emboli, ranging from conservative treatment to surgical intervention in severe cases.

## Introduction

Percutaneous vertebroplasty is a minimally invasive procedure widely used to manage vertebral compression fractures, particularly in elderly patients with advanced osteoporosis.^1^ Although generally effective and relatively safe, it can be complicated by cement leakage.^2,3^ Among such complications, intracardiac cement embolism is a rare but clinically significant adverse event that may go undetected without appropriate post-procedural imaging.

## Summary figure

**Figure:**
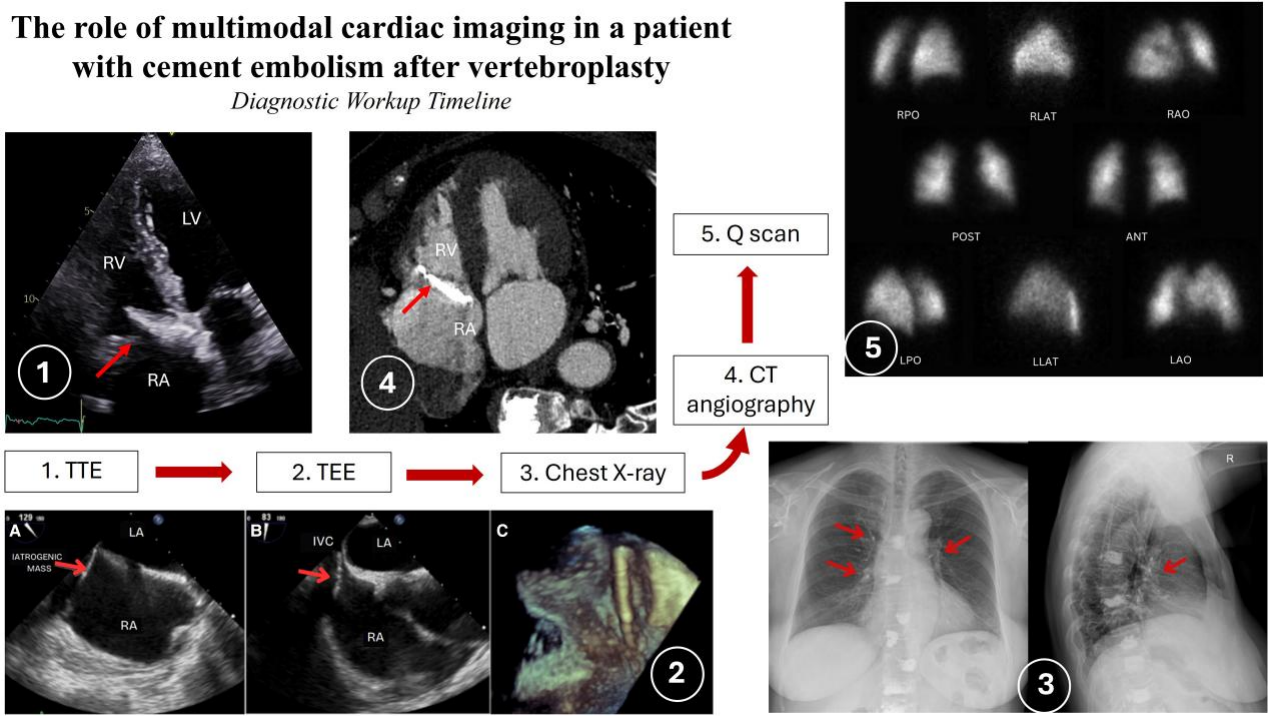


## Case presentation

A 67-year-old female patient was referred to our hospital with dyspnoea that had begun three months earlier. Her medical history included multiple vertebral fractures due to advanced osteoporosis, treated with vertebroplasty procedures at L1-L3 performed nine months prior. She also had a history of epilepsy and arterial hypertension. Upon admission, physical examination was unremarkable except for chest pain on palpation. Cardiovascular examination revealed normal blood pressure and a regular heart rate and rhythm. Peripheral pulses were symmetrical, and no peripheral oedema was present. All laboratory tests were normal, including high-sensitivity troponin I, brain natriuretic peptide, and D-dimers.

**Figure 1:**
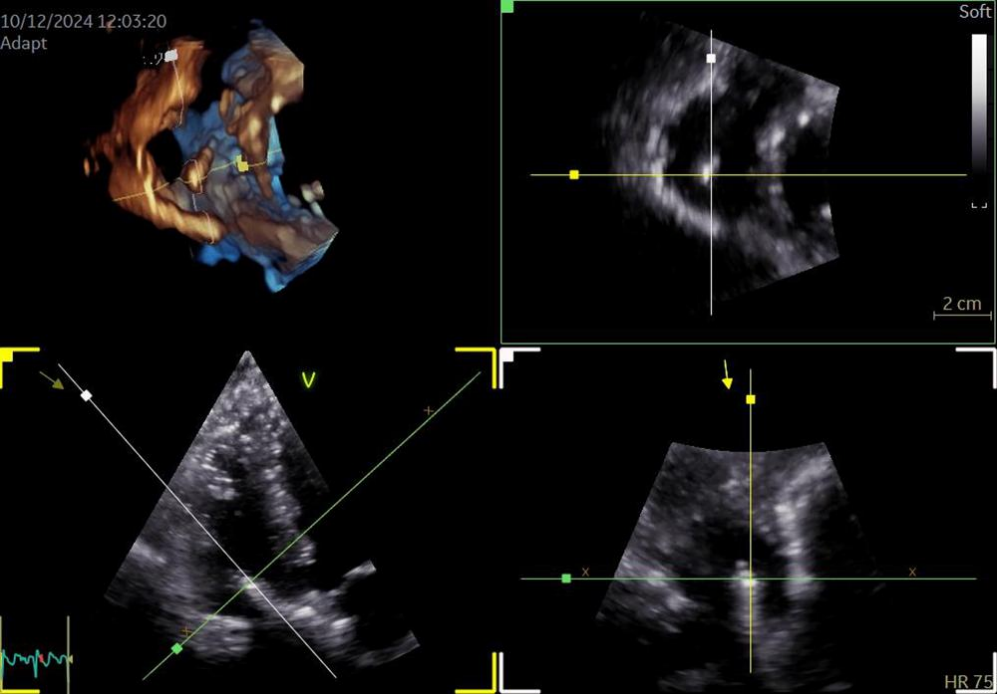
3D MPR images provide improved delineation of the mass, allowing more accurate assessment of its size, spatial location, and relationship to the surrounding cardiac structures, including the tricuspid valve. The detailed description of the imaging findings corresponds to that shown in Supplementary material online, *Video S1*.

Transthoracic echocardiography (TTE) with additional 3D images and 3D MPR (multiplanar reconstruction) (see Supplementary material online, *Videos S1* and *S2*, *Figure 1*) revealed an additional elongated mass within the right atrial cavity, near the tricuspid valve. The mass was motionless and positioned diagonally within the right atrium, extending from the atrial septum toward the anterior leaflet of the tricuspid valve. Left ventricular systolic function was normal, and moderate tricuspid regurgitation was noted.

**Figure 2:**
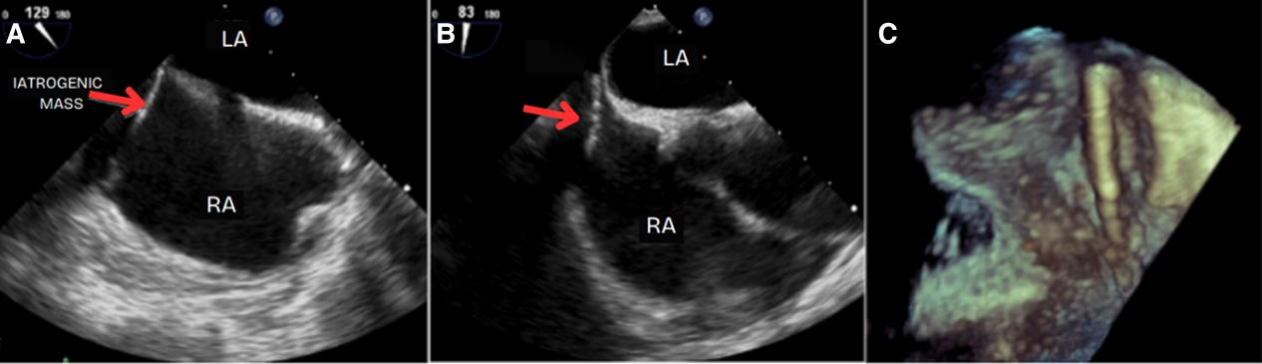
A transoesophageal echocardiography showing an elongated, rigid accessory mass, likely of iatrogenic origin, extending from the right atrium (RA) into the right ventricle in two-dimensional and three-dimensional images. LA, left atrium.

A transoesophageal echocardiography (TEE) was planned to assess the exact location of the mass and its relation to the tricuspid valve. However, the procedure was postponed due to additional vertebroplasty procedures at the thoracic vertebrae. Later that month, a TEE confirmed the presence of an elongated, rigid accessory mass (*Figure 2*). The mass originated in the right atrium, followed the atrial septum, extended through the tricuspid valve, and terminated in the right ventricle just below the tricuspid valve. Moderate regurgitation was observed near the foreign mass, and the estimated pulmonary artery systolic pressure was ∼40 mmHg.

**Figure 3:**
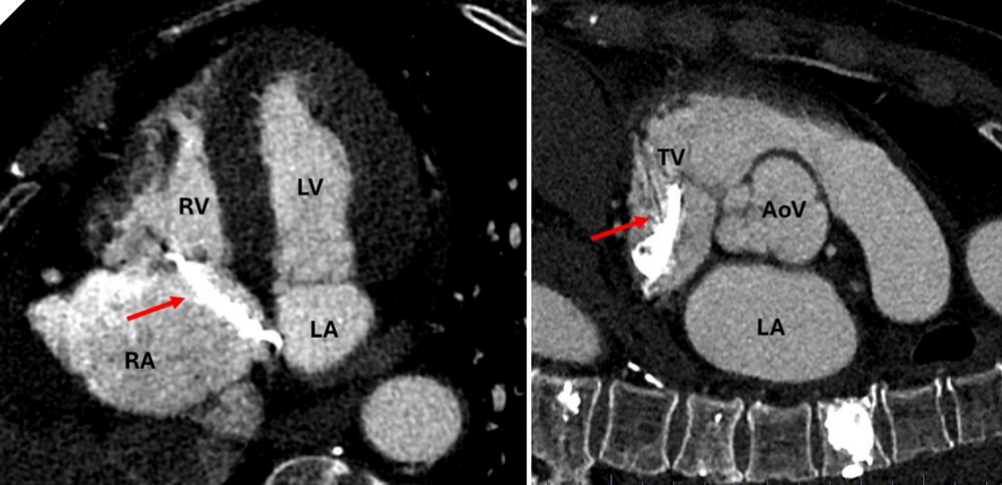
CT angiography multiplanar reconstructions showing the cement mass and its relation with cardiac structures. During post-processing, the projections were reformatted to match standard echocardiographic imaging planes, facilitating comparison and precise assessment of cardiac structures. AoV, aortic valve; LA, left atrium; LV, left ventricle; RA, right atrium; RV, right ventricle; TV, tricuspid valve.

Chest radiography revealed high-density embolic masses within the pulmonary vessels (see Supplementary material). Computed tomography angiography (CTa) demonstrated cement leakage into the paravertebral veins, beginning at the level of Th8. Cement was also present within several segmental branches of the pulmonary arteries. A hyperdense, approximately 60-mm-long and 6-mm-thick structure with multiple artefacts was visualized along the left inferior wall of the right atrium, extending into the coronary sinus. (*Figure 3*).

Q-scan imaging (see Supplementary material) demonstrated a subsegmental perfusion defect in the right S3 (anterior segment) with partial signs of reperfusion. Additional subsegmental perfusion defects were noted in the right S4 (lateral segment) and S8 (anterior basal segment), as well as bilateral apical (S1/2) perfusion disturbances, again with evidence of partial reperfusion.

Taken together, the imaging findings and the patient’s clinical history strongly suggested that both the intracardiac and pulmonary embolic masses represented cement emboli, a complication of previous vertebroplasty procedures. The case was discussed at a structural heart meeting with interventional cardiologists and cardiac surgeons, where percutaneous retrieval was deemed unfeasible due to anatomical challenges and the emboli’s proximity to the coronary sinus walls. Open-heart surgical extraction was considered high risk because embolic material was already present in both the cardiac chambers and the pulmonary circulation, and the cement fragments were friable, increasing the likelihood of further embolization during manipulation. After discussion with the patient, conservative management was continued. She remains under outpatient follow-up, with an uneventful clinical course to date.

## Discussion

Percutaneous vertebroplasty is a widely accepted and commonly performed minimally invasive procedure for treating osteoporotic or malignant vertebral compression fractures.^1^ The procedure involves the injection of polymethylmethacrylate cement into a damaged vertebral body, stabilizing it, relieving pain, and restoring its height.^4^ Percutaneous vertebroplasty is generally considered a safe procedure, with serious complications reported as low as 1.1–3.3%.^2^ On the other hand, cement leakage beyond the vertebral body is a relatively common occurrence, occurring in up to 54.7% of cases.^3^ While most cement leakages remain local, migration of a cement embolus from the vertebral venous plexus to the inferior vena cava, right heart chambers, or pulmonary arteries can occur, potentially leading to intracardiac or pulmonary embolism.

Cement pulmonary embolism is documented to occur in up to 6.8% of cases, while intracardiac embolism is seen in 3.9% of cases. However, the precise incidence of this unfavourable outcome is uncertain due to the absence of routine post-surgery chest imaging.^5,6^ Most cases have no clinical manifestation; however, the symptoms that are most reported include chest pain, dyspnoea, and palpitations.^6–9^ Nevertheless, severe conditions such as acute respiratory distress syndrome,^10^ cardiac tamponade,^11^ or perforation, which can lead to fatal outcomes, have also been reported.^8,12,13^ Manifestation of symptoms can vary from a few hours to years after the procedure has been completed.^6–13^ Cement emboli in the right atrium are usually asymptomatic or only mildly symptomatic, with no pericardial effusion and normal cardiac systolic function. In contrast, cement leakage reaching the right ventricle is more likely to result in pericardial effusion and cardiac wall perforation.^6,9^

Since TEE is considered the gold standard for imaging cardiac masses, in our case, we found 3D TTE to provide additional clinically meaningful information. Three-dimensional transthoracic imaging enabled comprehensive visualization of cardiac structures, offering a clearer depiction of the mass’s size, location, and spatial relationship with adjacent anatomy. Moreover, 3D imaging allowed real-time evaluation of the mass throughout the cardiac cycle, improving our understanding of its dynamic behaviour. Multiplanar reconstruction further enhanced diagnostic accuracy by enabling visualization of the mass in multiple orthogonal planes (sagittal, coronal, and transverse), allowing more detailed assessment of its extent and chamber involvement. Finally, 3D volumetric analysis proved essential for treatment planning, procedural considerations, and longitudinal monitoring.

CT is an indispensable modality in suspected cement embolization, given its excellent capability to detect high-density foreign material with high spatial resolution. Cardiac MRI may also be considered, as it provides complementary functional and structural information that is not attainable with other imaging techniques. In this case, CMR was not performed because its findings would not have altered the patient’s conservative management plan.

Although the European Society of Cardiology (ESC) guidelines do not specifically address intracardiac or pulmonary cement embolism, treatment strategies are often tailored to the severity of symptoms and the risk of complications. Asymptomatic cases can be managed without the need for anticoagulation, as research has demonstrated that the bone cement used in vertebroplasty does not induce thrombosis or platelet activation.^14^ Active intervention is recommended for symptomatic patients, which may include anticoagulation, percutaneous retrieval, or open-heart surgery to extract the emboli or in case of perforation.^7–9^

## Conclusions

Intracardiac cement embolism is a rare but clinically important adverse effect that can occur after percutaneous vertebroplasty. Although most cases may not have a clinical presentation, the symptoms can manifest months or years after the procedure. Multimodality imaging is essential for accurately assessing the cement’s location and its relationship to the surrounding structures. Clinicians should be attentive to the possibility of embolism development, especially in patients with a history of multiple vertebroplasty procedures.

## Lead author biography


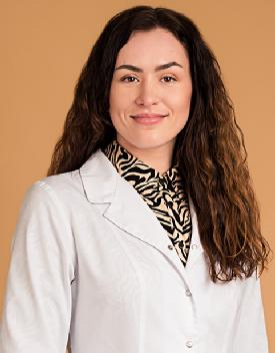
Eglė Majauskienė is a cardiologist and PhD student working in Vilnius University Hospital Santaros clinics. Her clinical and research interests focus on echocardiography and multimodality imaging, with an emphasis on improving the diagnostic accuracy and management of cardiovascular diseases.

## Supplementary Material

ytag263_Supplementary_Data

## Data Availability

The data underlying this article are available in the article and in its online Supplementary material.
