# Supplementary material for: From spine to heart: a case report of massive cement embolism following vertebroplasty
Source: Eur Heart J Case Rep. 2026 Apr 15;10(5):ytag263. doi: 10.1093/ehjcr/ytag263 (PMC13158959; doi:10.1093/ehjcr/ytag263)
Supplement: ytag263_Supplementary_Data [file ytag263_supplementary_data.zip › Supplementary material.docx]

Supplementary material


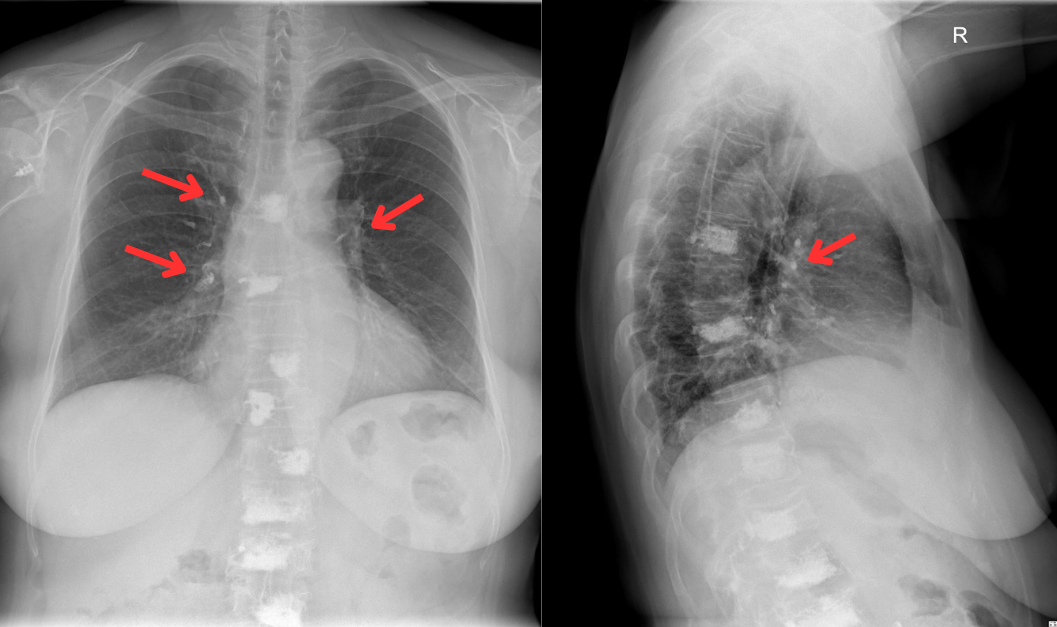


**Figure 1.** Chest X-ray showing multiple small cement emboli in the pulmonary vessels (segmental arteries) on both sides, with emboli slightly more pronounced on the right.


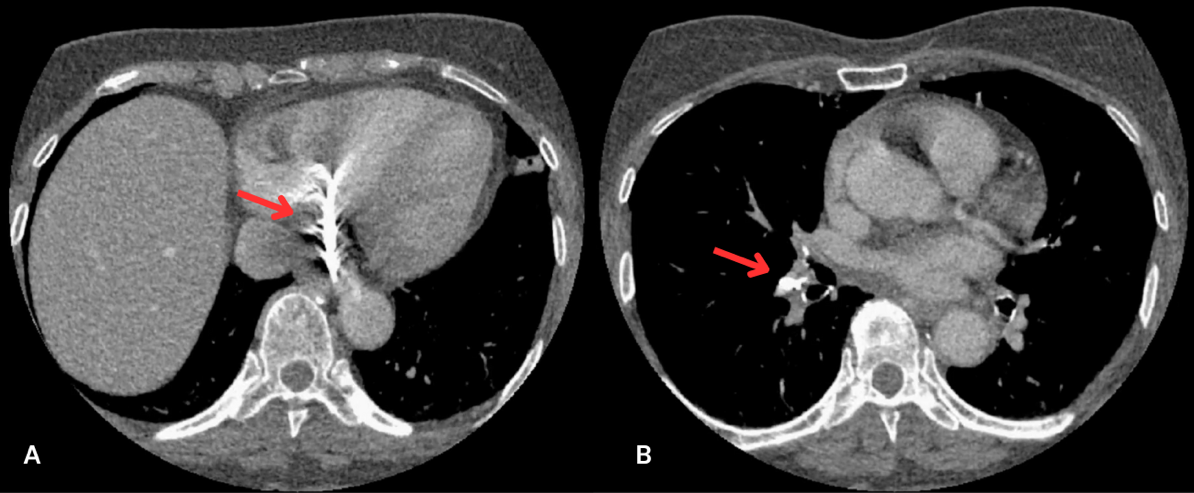


**Figure 2.** Computed tomography angiography findings: A hyperdense structure with extensive artifacts is observed along the left inferior wall of the right atrium, extending into the coronary sinus (A, arrow). Additionally, hyperdense masses are noted in the pulmonary artery branches (B, arrow).


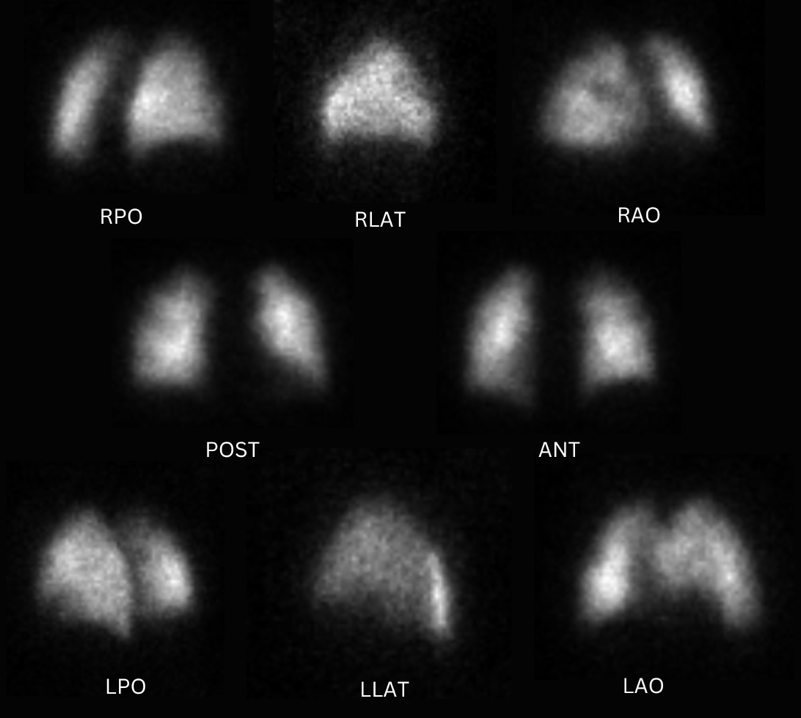


**Figure 3**. Eight-view of lung perfusion scintigraphy demonstrating scintigraphic evidence of small-scale chronic pulmonary embolism (PATE), with most areas showing partial signs of reperfusion. RPO = right posterior oblique; LPO = left posterior oblique; RAO = right anterior oblique; LAO = left anterior oblique; RLAT= right lateral; LLAT= left lateral; ANT= anterior; POST= posterior.
