# Supplementary material for: From spine to heart: a case report of massive cement embolism following vertebroplasty
Source: Eur Heart J Case Rep. 2026 Apr 15;10(5):ytag263. doi: 10.1093/ehjcr/ytag263 (PMC13158959; doi:10.1093/ehjcr/ytag263)
Supplement: ytag263_Supplementary_Data [file ytag263_supplementary_data.zip › Video captions.docx]

**Video 1.** Reconstructed 3D TTE view mimicking a short-axis projection of the atrioventricular valves. The tricuspid valve is visualized in the upper left, and the mitral valve on the right. A hyperdense wire-like structure is visualized protruding from the right atrium into the right ventricle, occupying the inflow portion of the RV.

**Video 2.** Three-dimensional multiplanar reconstruction (3D MPR) demonstrates a hyperechogenic, non-mobile, elongated mass measuring approximately 5 cm in maximal length, located adjacent to the atrial septum. The mass is oriented diagonally within the right atrium and extends across the tricuspid valve into the right ventricle, approaching the anterior leaflet of the tricuspid valve.
